# La maladie de Hoffman ou perifolliculitis capitis abscedens et suffodiens

**DOI:** 10.11604/pamj.2013.14.151.2510

**Published:** 2013-04-16

**Authors:** Hayat Bourra, Badreddine Hassam

**Affiliations:** 1 Service de Dermatologie, CHU Ibn Sina, Université Med V, Souissi, Rabat, Maroc

**Keywords:** Maladie de Hoffman, cellulite disséquante, alopécie cicatricielle, Hoffman disease, dissecting cellulitis, scarring alopecia

## Image en médicine

La maladie de Hoffman ou perifolliculitis capitis abscedens et suffodiens dite cellulite disséquante du cuir chevelu est une cause rare d'alopécie cicatricielle, faisant partie des folliculites décalvantes. Il s'agit d'une suppuration profonde chronique, non infectieuse, dont l’étiologie est inconnue atteignant préférentiellement les hommes entre 20 et 40 ans, noirs dans 80% des cas. Les lésions forment des nodules profonds et des collections purulentes communiquant entre elles en formant des sinus, le tout sous un cuir chevelu inflammatoire douloureux et alopécique. En dermoscopie Les orifices folliculaires apparaissent dilatés comme de gros points jaunes, coalescents en bulles de savon. L'association à une acné conglobata, à une hidrosadénite suppurée ou à des kystes pilonidaux a été rapportée. Une étiopathogénie commune à ces atteintes a été soulevée par occlusion folliculaire, rupture folliculaire puis infection secondaire. Plusieurs thérapeutiques ont été essayées, l'isotrétinoïne apparait comme le traitement de choix. Nous rapportons le cas d'un patient de 21 ans, sans antécédents pathologiques particuliers, qui consulte pour des nodules douloureux du cuir chevelu évoluant depuis plus de 6 mois, sans notion de fièvre ni de traumatisme. L'examen clinique trouve plusieurs nodules sous cutanés fluctuants de diamètre variant de 0,5 à 2cm de grand axe, sensibles à la palpation disséminés au niveau du vertex, associés à quelques pustules. On notait par ailleurs une acné avec quelques lésions nodulo kystiques du visage. L'examen bactériologique du prélèvement de pus au niveau d'un nodule était stérile. La biopsie cutanée était non spécifique. Le patient fut mis sous isotrétinoine à la dose de 0,5mg/kg/j avec bonne évolution.

**Figure 1:**
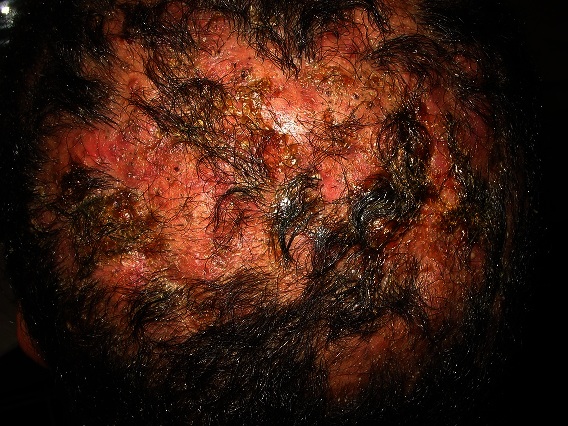
Nodules inflammatoires et douloureux confluents entre eux, formant des sinus au niveau du vertex et de l'occiput associés à quelques pustules

